# DNA Barcodes for Nearctic Auchenorrhyncha (Insecta: Hemiptera)

**DOI:** 10.1371/journal.pone.0101385

**Published:** 2014-07-08

**Authors:** Robert G. Foottit, Eric Maw, P. D. N. Hebert

**Affiliations:** 1 Agriculture and Agri-Food Canada, Invertebrate Biodiversity – National Environmental Health Program, and Canadian National Collection of Insects, Arachnids and Nematodes, Ottawa, Ontario, Canada; 2 Biodiversity Institute of Ontario, University of Guelph, Guelph, Ontario, Canada; The New York Botanical Garden, United States of America

## Abstract

**Background:**

Many studies have shown the suitability of sequence variation in the 5′ region of the mitochondrial cytochrome *c* oxidase I (COI) gene as a DNA barcode for the identification of species in a wide range of animal groups. We examined 471 species in 147 genera of Hemiptera: Auchenorrhyncha drawn from specimens in the Canadian National Collection of Insects to assess the effectiveness of DNA barcoding in this group.

**Methodology/Principal Findings:**

Analysis of the COI gene revealed less than 2% intra-specific divergence in 93% of the taxa examined, while minimum interspecific distances exceeded 2% in 70% of congeneric species pairs. Although most species are characterized by a distinct sequence cluster, sequences for members of many groups of closely related species either shared sequences or showed close similarity, with 25% of species separated from their nearest neighbor by less than 1%.

**Conclusions/Significance:**

This study, although preliminary, provides DNA barcodes for about 8% of the species of this hemipteran suborder found in North America north of Mexico. Barcodes can enable the identification of many species of Auchenorrhyncha, but members of some species groups cannot be discriminated. Future use of DNA barcodes in regulatory, pest management, and environmental applications will be possible as the barcode library for Auchenorrhyncha expands to include more species and broader geographic coverage.

## Introduction

The hemipteran suborder Auchenorrhyncha includes two large superfamilies, Fulgoroidea (planthoppers) and Membracoidea (treehoppers and leafhoppers), and two smaller superfamilies, Cercopoidea (spittlebugs or froghoppers) and Cicadoidea (cicadas). In North America north of Mexico, there may be as many as 3800 species of Auchenorrhyncha [1]–[4]. The Nearctic component of the Mexican fauna is poorly known but may be equally as rich. Many species of Auchenorrhyncha, especially leafhoppers, are economically important as either direct plant pests or as vectors of plant pathogens [5]. Some tree, shrub and grass-feeding Auchenorrhycha are host specific [6]–[8], and leafhoppers have been used as indicators of habitat quality, particularly in grasslands where they are especially diverse [8], [9].

Sequence variation in the 5′ end of the mitochondrial cytochrome *c* oxidase subunit I gene (COI) has been adopted as the DNA barcode for the identification of species in the animal kingdom [10], [11]. DNA barcode data are already available for several groups of Hemiptera (Aphididae [12], [13], Adelgidae [14], Heteroptera [15], [16], Coccoidea [17]). However, very little sequence information is available for the barcode region in Auchenorrhyncha. The only broad surveys are those of Kamitani [18], who provided DNA barcodes for 45 species of Japanese Cicadellidae, Cryan and Svenson [19] who included COI sequences for 80 species as part of their investigation of family-level relationships among Cercopoidea, and Lin & Wood [20] in a study of tribal relationships and the evolution of maternal care in Membracinae. In addition, several genus-level or species-group phylogenetic analyses and investigations of population variation have included all or part of the barcode region [21]–[32] providing intensive within-species replication. Two other studies employed COI barcodes to identify cicadellid prey items [33]–[34] while Le Roux and Rubinoff [35] used COI sequences to help determine the source of a leafhopper adventive to Hawaii. Seabra *et al.* [36] examined the use of ‘barcoding’ in *Philaenus*, but used the 3′ end of the COI gene, generating results that are not directly comparable to the global standard.

The present study provides a preliminary library of COI barcodes for Nearctic Auchenorrhyncha, primarily from Canada and the northern United States, based on material in the Canadian National Collection of Insects, Arachnids and Nematodes (Agriculture and Agri-Food Canada, Ottawa) (CNC).

## Materials and Methods

### Specimens

A total of 1482 dried, pinned specimens of Cicadidae, Cercopidae (including Aphrophoridae), Cicadellidae, Membracidae and Aetalionidae were selected from the CNC (Table 1). Most of the specimens were larger-bodied forms collected by hand so several cicadellid subfamilies, consisting mainly of species with small body size were not represented. The majority of the species were from North America north of Mexico (including several introduced species), but material from other areas was examined for certain groups. An average of 2.5 specimens was selected per species, chosen when practical to provide geographic coverage. The age of specimens ranged from one to 60 years. All specimens of leafhoppers and spittlebugs, as well as many of the treehoppers were determined, or the identification was confirmed, by KGA Hamilton. For other groups, identifications applied to the specimens by past workers were assumed to be correct. In some cases, narrow generic concepts were applied to assess the utility of COI barcodes in assigning generic names to species not represented in the data set (see Table 1). Names of genera and higher taxa of leafhoppers follow Oman [2]; higher classification of spittlebugs follows the Metcalf Catalog [37], while the generic names are based on the work of Cryan and Svenson [19] and Hamilton [38]. Names for other taxa follow the most recent checklists [3], [4], [39], [40]. Authorship of generic and specific names is provided in these references.

**Table 1:** Genera and number of barcoded species in each auchenorrhynch family group. Non-Nearctic genera are designated by an asterisk (*).

Number of species examined/genus	5 or more (number of species indicated)	4	3	2	1
Cicadidae	*Okanagana* (16), *Tibicen* (nearctic spp.) (12)		*Platypedia*	*Cicadetta, Diceroprocta, Pacarina,*	Beameria, Cacama, *Cicada, Neocicada, Quesada, *Tibicen (Japan)
Fulgoridae and Dictyopharinae	*Alphina* (4), *Cyrpoptus* (5), *Scolops* (13)		*Rhynchomitra*	*Acraephia, Amycle, *Calyptoproctus, Phylloscelis, Poblicia	Alaruasa, *Copidocephala, *Hypaepa, Nersia
Aphrophoridae and Cercopidae	*Lepyronia* (5), *Plesiommata* 1 (12)		*Cephisus, Philaenarcys*	*Philaronia, Prosapia*	Aphrophora, *Jembrana, *Neophilaenus, Paraphilaenus, *Philaenus, Xenaphrophora, *Yunnana
Cicadellidae	*Cuerna (22), Draeculacephala* (26), *Errhomus* (21), *Evacanthus* (7), *Graphocephala* (8), *Gyponana* (20), *Hordnia* (5), *Keonolla* (7), *Ponana* (11), *Prairiana* (8), *Rugosana* (7), *Xerophloea* (7)	*Carsonus, Hamana, Hecalus, Helochara, Marganalana* 2, *Memnonia, Momoria* 3, *Tylozygus*	*Acusana, Attenuipyga, Dorycara, Homaldisca, Marganana, Negosiana, Neokolla, Oncometopia, Penestragania* 3, *Phera	*Ciminius, Hylaius, Neohecalus, Pagaronia, Paraulacizes, Provancherana, Similitopia, Spangbergiella, Thatuna*	*Apogonalia, *Bascarrhinus, Colimona, Decua, Dragonana, Dicyphonia, *Gypona, Hortensia, Lystridea, Polana, *Proranus, *Titiella
Membracidae and Aetalionidae	*Entylia* (7), *Enchenopa* (9), *Glossonotus* (5), *Heliria* (5), *Ophiderma* (6), *Stictocephala* (10) *Stictopelta* (5), *Telamona* (21), *Vanduzeea* (5)	*Atymna, Campylenchia, Cyrtolobus, Spissistilus, Tortistylus, Tylocentrus*	*Anisotylus, Carynota, Centrodontus, Grandilobus, Micrutalis, Publilia, Smilia, Trasymedes*	*Acutalis, Ashmedea, Centrodontus, Gargara, Microcentrus, Philya, Platycentrus, Platycotis, Telamonanthe, Trachytalis, Tylopelta,*	*Aconophora, Aetalion, Antianthe, Archasia, Atymnina, Bajulata, *Bolbonota, Bryantopsis, *Ceresa,*Cyphonia, *Enchophyllum, Hadrocephala, Hypsoprora, Idioderma, Leioscyta, *Membracis, Micrutalis, Parantonae, *Phyllotropis, Polyglypta, *Poppea, Scalmorphus, Thelia,Trichaetipyga, Umbonia

1) often treated as a subgenus of *Aphrophora*.

2) often treated a subgenus subgenus of *Gypona*.

3) generic segregates of *Stragania*.

Sequences, trace files, collection data, and specimen photographs are deposited in the Barcode of Life Data Systems (BOLD, http://www.boldsystems [41]) as public dataset DS-EMAUCH0 (dx.doi.org/10.5883/DS-EMAUCH0). Sequences are also available in GenBank (accession numbers KF919304 - KF920463). A label was added to each specimen enabling its linkage with the corresponding record in BOLD.

### COI Amplification, Sequencing and Analysis

DNA was extracted from a single leg (left middle leg whenever possible) removed from each specimen. Extraction, PCR amplification and sequencing were performed at the Biodiversity Institute of Ontario (Guelph, Ontario, Canada) following procedures described in Hajibabaei et al. [42] Primer names and sequences are given in Table 2. If the first pass using the pair LepF-t1/LepR failed, further reactions were performed using internal primers or cocktails of mixed primers.

**Table 2:** PCR primers used in this study.

Primer Name	Primer sequence (5′-3′)	direction	Primer source
LepF2_t1	M13Fb)-AATCATAARGATATYGG	F	Modified from [54]
LepF1	ATTCAACCAATCATAAAGATATTGG	F	[54]
LepR1	TAAACTTCTGGATGTCCAAAAAATCA	R	[54]
LCO1490	GGTCAACAAATCATAAAGATATTGG	F	[55]
HCO2198	TAAACTTCAGGGTGACCAAAAAATCA	R	[55]
tRWF1_t1	M13Fb)-AAACTAATARCCTTCAAAG	F	[56]
tRWF2_t1	M13Fb)-AAACTAATAATYTTCAAAATTA	F	[56]
C_tRWFa)	tRWF1_t1+tRWF2_t1	F	[56]
MLepF1	GCTTTCCCACGAATAAATAATA	F	Paul Hebert
MLepR2	GTTCAWCCWGTWCCWGCYCCATTTTC	R	Sean Prosser
C_LepFolFa)	LepF1+LCO1490	F	Natalia Ivanova
C_LepFolRa)	LepR1+HCO2198	R	Natalia Ivanova
MHemF	GCATTYCCACGAATAAATAAYATAAG	F	[16]
MHemR	GGTGGATAAACTGTTCAWCC	R	[16]

The same primers were used for sequencing amplicons, except that M13F was used to sequence strands primed with the M13-tailed primers.

a) cocktail primers: indicated primers combined.

b) M13F = TGTAAAACGACGGCCAG.

Sequences were assembled and edited using CodonCode Aligner Ver2.0.6 (CodonCode Co.). Pairwise divergences were calculated using both uncorrected values and the Kimura two-parameter (K2P) model of base substitution [43], and several other substitution models were explored for various subsets of the data. Athough K2P is not necessarily the best model to employ [44], [45], the values derived from this model are reported here for generic and family level summaries because it allows for direct comparison with the existing COI barcode literature for Hemiptera [12]–[18]. For the smaller divergence values encountered within species and among closely related species, the difference among models (including uncorrected distance) was small, often less than the reporting precision, and model choice did not affect the conclusions drawn. Both K2P and uncorrected values are given for species-level comparisons when they differ; when no model is specified there was no difference at the reported precision. Summary statistics were calculated using the utilities available in BOLD. Additional analysis of base substitution rates and exploration of alternate substitution models was carried out using MEGA version 5 [46].

## Results

Summary statistics are given in Table 3. Results are reported separately for the following groups: Fulgoroidea, Cicadidae, Cercopoidea (Aphrophoridae, Cercopidae, Clastopteridae), Cicadellidae, and Membracidae plus Aetalionidae. Of the 1482 specimens analyzed, 1150 (77%, representing 471 identified species and 66 undetermined OTUs) produced sequences longer than 400 base pairs. Nearly 75% of these (870 of the 1150) met the barcode standard (at least 500 base pairs, less than 1% ambiguous residues, bidirectional sequence coverage [47]). One sequence with a single base pair deletion was assumed to represent a possible NUMT and was excluded. A second sequence was excluded, pending replication, as possible contamination, since the nearest matches were for non-hemipterans. Several sequences with significant background signal from co-amplified products were also excluded. Sequences less than 400 base pairs in length were not included in subsequent analyses, but are available on BOLD (data set DS-EMAUCH1, dx.doi.org/10.5883/DS-EMAUCH1) as many of these records provide the sole coverage currently available for the taxa in question.

**Table 3:** Summary statistics for 1150 sequences recovered from museum specimens of Auchenorhyncha.

	Fulgoridae	Cicadidae	Cercopoidea(a)	Cicadellidae	Membracidae(b)	total
number of specimens submitted	86	47	123	762	464	1482
age of specimens, range (mean) in yr	1–56 (35)	8–56 (33)	1–81 (24)	1–57 (28)	3–59 (35)	
success rate (seq >399 bp/specimens sampled)	65%	93%	88%	79%	72%	77%
proportion of sequences barcode compliant	62%	65%	81%	79%	67%	75%
age of specimens with sequence >399 bp	1–56 (32)	8–56 (34)	1–52 (23)	1–56 (25)	3–59 (32)	
No. of specimens with sequences >399 bp	56	44	109	606	335	1150
>600 bp, (barcode compliant/total)	35/35	27/29	81/85	470/471	219/221	837/841
500–600 bp, (barcode compliant/total)	0	2/3	8/8	16/16	7/7	33/34
400––499 bp	20	12	16	119	107	275
no. of identified species	30	41	32	231	137	471
with >2 specimens	6	0	25	115	49	195
with 2 specimens	10	2	4	60	34	110
with 1 specimen	14	39	3	56	54	166
no. of undetermined OTUs(c)	0	1	1	37	27	66
with >2 specimens	0	0	0	9	3	12
with 2 specimens	0	0	0	5	3	8
with 1 specimen	0	1	1	23	21	46
intraspecific divergence(e) (%) (K2P)						
range (K2P)	0–2.29	0–1.01	0–7.47	0–8.26	0–5.28	0–8.26
mean ± se (K2P)	0.36±0.02	0.50±0.25	0.51±0.04	0.55±0.002	0.64±0.01	0.56±0.03
range (uncorrected)	0–2.24	0–1.01	0–7.03	0–7.71	0–4.98	0–7.71.
mean ± se (uncorrected)	0.36±0.02	0.50±0.25	0.50±0.04	54±0.002	0.63±0.01	0.55±0.03
distance^e^ to nearest neighbor (% of species)						
minimum pairwise distance <1%	10%	26%	37%	28%	19%	25%
minimum distance 1 to 2%	13%	7%	0%	4%	3%	4%
minimum distance >2%	76%	65%	62%	67%	79%	70%
no. of genera	11	11	10	59	56	147
no. of genera with >1 species(d)	6	5	5	40	32	88
interspecific divergence within genus (K2P,%)						
range	0.15–16.5	0.15–23.25	0–20.465	0–26.81	0–26.19	0–26.81
mean ± se	8.87±0.01	9.33±0.02	8.28±0.004	9.82±0.001	11.69±0.004	9.91±0.06
inter-generic divergence within family (K2P,%)						
range	7.9–28.9	14.6–30.9	10.0–29.5	5.1–40.2	0.2–43.0	0.2–43
mean ± se	19.0±0.2	22.3±0.0	19.4±0.0	24.6±0.0	24.3±0.0	24.5±0.01

a) Aphrophoridae and Cercopidae.

b) Membracidae including Aetalionidae.

c) undetermined at species level, including undescribed taxa.

d) includes undetermined/undescribed species.

e) normalized for number of specimens per species.

Although one specimen collected 60 years ago yielded a full sequence, the probability of obtaining results from dry specimens declined with age, dropping from 98% recovery in specimens analyzed within a decade of capture to 57% in specimens more than 50 years old.

The LepF-t1 primer was less effective than the other forward primers. As a result, many sequences only provided coverage for about 400 bp at the 3′ end of the barcode region (i.e. upstream of primer MHemF or MLepF primers, closely positioned to the primer ‘Ron’ or C1-J-1718, often used in phylogenetic studies e.g. [19], [29]. The proportional contribution of each base change to the total divergence values for these truncated sequences is slightly inflated. As well, analysis indicated that site changes were more frequent in this region, and pairwise K2P distances in the 400 bp at the 3′ end of the barcode region averaged 1.08 times higher than those for the full length of the same sequence.

In general, both mean (0.36%–0.64% K2P or 0.36–0.63% uncorrected; Table 3) and maximum intraspecific divergences were low. For example, only 22 (Table 4) of the 304 species with more than one specimen possessed a maximum K2P divergence greater than 2%. In contrast to these general patterns, there were some groups in which between-species and within-species sequence variation at COI showed little or no discontinuity, i.e. the species lacked a barcode gap. Overall, the minimum nearest neighbor distance was less than 1% in 10%–37% of the species (25% average) in the various families (Table 2) and another 0%–13% (4% average) of species showed just 1 to 2% K2P sequence divergence. Table 5 lists the species groups with minimum pairwise K2P divergence of less than 2%.

**Table 4:** Species with maximum intraspecific pairwise divergence (K2P) greater than 2%.

	number of specimens	K2P distance (range, %)	uncorrected distance (range, %)
**Aphrophoridae**			
*Cephisus variolosus*	3	3.47–7.47	3.37–7.03
**Cicadellidae**			
*Carsonus aridus*	3	0.15–2.82	0.15–2.76
*Carsonus furcatus*	3	0–8.26	0–7.71
*Draeculacephala soluta*	3	0.15–2.18	0.15–2.14
*Errhomus josephi*	3	0–3.30	0–3.21
*Errhomus lineatus*	3	0–7.72	0–7.21
*Graphocephala coccinea*	2	2.02	1.99
*Gyponana extenda*	3	0.15–2.02	0.15–1.99
*Gyponana hasta*	3	2.99–4.77	2.91–4.59
*Gyponana praelonga*	2	2.42	2.37
*Gyponana quebecensis*	3	1.40–2.34	1.22–2.29
*Keonolla uhleri*	2	2.02	1.99
*Oncometopia orbona*	3	0–2.66	0–2.60
*Pagaronia minor*	3	3.77	3.67
*Pendarus punctiscriptus*	2	2.03	1.99
*Rugosana fibrata*	3	1.00–3.14	1.00–3.06
**Membracidae**			
*Carynota stupida*	5	0.15–2.27	0.15–2.24
*Echenopa binotata*	2	2.30	2.26
*Spissistilus festinus*	5	0.25–2.57	0.25–2.50
*Stictocephala albescens*	2	2.04	2.00
*Telamona tristis*	6	0.75–3.09	0.75–3.01
*Telamonanthe pulchella*	6	2.54–5.23	2.49–4.98
*Tylocentrus quadricornis*	2	3.15	3.05

**Table 5:** Groups of species with low interspecific distance (minimum pairwise K2P divergence among members of group is less than 2%).

	K2P distance (range, %)	separation criteria
**Cicadidae**		
*Cicadetta caliope/kansa*	0.25	
*Okanagana bella/canescens/rubracaudata*	0.15–0.48	
*Okanagana canescens/rubracaudata*	0.15	
*Okanagana bella/canescens*	0.48	
*Okanagana lurida/occidentalis*	0.16	
*Okanagana utahensis/vanduzeei/villosa*	0.55–1.4	
*Okanagan vanduzeei/villosa*	0.55	
*Okanagana utahensis/vanduzeei*	1.4	
*Tibicen auletes/resonans*	0.75	
*Tibicen canicularis/linnei*	1.99	
**Aphrophoridae**		
*Aphrophora gelida/maculosa/permutata*	0–1.81	
*Aphophora princeps/regina*	0–0.31	
*Philaenarcys bilineata/killa/spartina*	0–1.71	
*Philaronia abjecta/canadensis*	0.46–0.75	
*Prosapia bicincta/ignipectus*	0–1.26	male genitalia; color pattern
**Cicadellidae**		
*Attenuipygia joycei/vanduzeei*	0	size, color
*Attenuipygia minor/platyrhynchus*	1.55	
*Bandara curvata/johnstoni*	0.75	
*Bonneyana schwartzi/terminalis*	0–0.15	male genitalia
*Cuerna alba/arida/balli*	0–0.5	male and female genitalia
*Cuerna angustata/flavipes/semibulba*	0.5–1.77	
*Cuerna cuesta/nielsoni/striata*	0–1.01	
*Cuerna nielsoni/cuesta*		geographic ssp.
*Draculaecephala antica/constricta/navicula/savannahae*	0.15–1.87	
*Draculaecephala antica/constricta*	0.15–0.77	
*Draculaecephala (antica+constricta)/savannahae*	1.51–1.87	
*Draculaecephala navicula/savannahae*	1.51	
*Draculaecephala borealis/bivoltina/crassicornis*	0–0.93	biology
*Draculaecephala borealis/bivoltina*	0–0.62	geographic ssp.
*Draculaecephala novaboracensis/prasina*	0.15–0.97	geographic ssp.
*Draculaecephala zeae/(robinsoni+sphagneticola)*	1.86	
*Draculaecephala robinsoni/sphagneticola*	0.15–0.62	color, size
*Draculaecephala paludosa/portola/zeae*	0.62–1.39	
*Draeculacephala angulifera/manitobiana*	0.33–0.99	geographic ssp.
*Errhomus paradoxus/winquatt*	1.51–1.77	
*Errhomus sobrinus/satus*	0	
*Evacanthus nigramericanus/orbitalis*	0.31–1.86	geographic ssp.
*Graphocephala (picta+teliformis)/fennahi/coccinea*	1.39–1.84	
*Graphocephala picta/teliformis*	0	biology, size
*Gyponana amara/aculeata*	0.62–0.85	
*Gyponana acia grp* *	0–3.29	wing venation, colour, biology, male genitalia
*Gyponana acia grp* * */vasta*	0.93–1.99	
*Gyponana acia grp* * */avara*	1.08–1.96	
*Gyponana octolineata/tubera/palma*	0.31–1.87	wing venation, colour, biology, male genitalia
*Gyponana aculeata/gladia*	1.71	
*Gyponana aculeata/cana*	1.71	
*Helochara delta/communis*	0.15–0.46	character displacement in size; hosts differ
*Keonolla confluens/surcula*	0.15–0.92	geographic ssp.
*Margalana contana/melanota*	0.15–0.62	geographic ssp.
*Neohecalus lineatus/magnificus*	0.93	
*Stragania alabamensis/apicalis*	0.46	
**Membracidae**		
*Acutalis brunnea/tartarea*	0–1.55	
*Campylenchia curvata*	0.31–0.77	
*Echenopa brevis/sp. nov. 1*	0.31–0.67	hosts
*Echenopa binotata/sp. nov. 2*	1.51–2.27	hosts
*Entylia carinata/emarginata*	0.47–1.24	
*Gargara genistae/nigromaculata*	1.27	
*Heliria praealta/Telemona colina*	1.68–2.34	
*Publilia brunnea/modesta*	0–1.39	
*Smilia camelus/fasciata*	1.26	
*Telamona barbata/pyramidata/spreta/viridia*	0–1.77	
*Tortistilus minutus/pacificus*	0–1.70	

*Gyponana acia grp = G. acia/cacuminal/extenda/mali/parallela/pingua/praelonga/quebecensis/salsa/serpent/striata. Within the *acia* group: max intraspecific distance = 2.42%; mean between species distance = 1.14%.

For genera represented by more than one species, the nearest neighbor (as measured by minimum pairwise K2P distance among specimens of each species) was usually a congeneric species so members of a genus were cohesive. However, 47 exceptions were detected (Table 6) with the membracid *Heliria* particularly noteworthy, as four of its five members had nearest- neighbors in another genus.

**Table 6:** Species belonging to genera represented by more than one species for which nearest neighbor is in a different genus.

species>nearest neighbor	minimum divergence (%)
**Cicadidae**	
*Tibicen townsendi>Cacama valvata*	16%
**Aphrophoridae:**	
*Lepyronia gibbosa>Cephisus variolosus*	14%
*Paraphilaenus notatus>Philaenarcys killa*	10%
*Paraphilaenus parallellus>Philaenarcys spartina*	8%
**Cicadellidae:**	
*Graphocephala psephena>Cuerna hasbroucki*	14%
*Graphocephala rufimargo>Keonolla sp.*	11%
*Graphocephala versuta>Keonolla lugubris*	15%
*Homalodisca elongata>Phera insolita*	10%
*Hordnia atropunctata>Homalodisca elongata*	13%
*Hordnia aurora>Phera insolita*	14%
*Hordnia cythura>Keonolla sp.*	13%
*Hordnia ignava>Keonolla lugubris*	13%
*Oncometopia nigricans>Similitopia sp.*	10%
*Margalana vexana>Gyponana aculeata*	14%
*Phera centrolineata>Homalodisca elongata*	10%
*Phera insolita>Homalodisca elongata*	10%
*Similitopia alpha>Cuerna cuesta*	13%
*Similitopia rufipennis>Cuerna obesa*	13%
*Similitopia sp. nov.>Oncometopia clarior*	10%
**Membracidae:**	
*Atymna distincta>Cyrtolobus inaequalis*	8%
*Atymna helena>Cyrtolobus puritanus*	9%
*Carynota marmorata>Telamona tristis*	5%
*Carynota stupida>Telamona tristis*	5%
*Cyrtolobus acutus>Atymna distincta*	12%
*Cyrtolobus inaequalis>Atymna distincta*	8%
*Cyrtolobus semifascia>Smilia fasciata*	8%
*Enchenopa apicalis>Campylenchia rugosa*	19%
*Enchenopa permutata>Leioscyta ferruginipennis*	9%
*Enchenopa sericea>Tylopelta gibbera*	17%
*Glossonotus acuminata>Heliria cristata*	∼0%
*Glossonotus turriculata>Telamona tristis*	9%
*Glossonotus univittata>Telamona tristis*	7%
*Grandolobus grandis>Atymna distincta*	12%
*Heliria clitella>Telamona tremulata*	7%
*Heliria cristata>Glossonotus acuminata*	∼0%
*Heliria gemma>Telamona gibberata*	8%
*Heliria molaris>Telamona fitchi*	1%
*Micrutalis dorsalis>Cyrtolobus inaequalis*	19%
*Ophiderma mus>Cyrtolobus semifascia*	13%
*Ophiderma pallida>Atymnina elongata*	12%
*Telamona concava>Glossonotus acuminata*	2%
*Telamona fitchi>Heliria molaris*	1%
*Telamona salvini>Cyrtolobus inaequalis*	17%
*Telamona tristis>Carynota marmorata*	5%
*Telamona unicolor>Carynota stupida*	7%
*Tortistilus inermis>Stictocephala brevicornis*	9%
*Tortistilus wickhami>Stictocephala brevicornis*	9%
*Vanduzeea arquata>Ashmeadea carinata*	13%

K2P divergence value rounded to nearest one percent.

## Discussion

Previous studies have provided DNA barcodes for only a few species of Auchenorrhyncha. Seabra et al. [36] found that COI sequences clearly discriminated European members of the genus *Philaenus* (Cercopoidea), while Le Roux & Rubinoff [35] used COI barcode sequences to identify the geographic origin of populations adventive to Hawaii in the leafhopper genus *Macrosteles*. Kamitani [18] provided DNA barcodes for 45 Japanese species of Cicadellidae. Although cercopids and membracids are well-represented as a result of broad phylogenetic analyses [19], [20], this study provides the first large-scale data release of COI barcode records for the suborder, with representation of about 8% of the known species of Auchenorrhyncha found in Canada and the United States. In fact, 8 of the 13 species of Fulgoroidea and 46% of the species of Cercopoidea present in this region were barcoded.

The mean intraspecific sequence divergence was less than 0.7% for the species examined in this study, but future work may increase this value because sample sizes are small and geographic coverage is limited for most species. These values are similar to those reported for Heteroptera (0.74% intraspecific, 10.67% interspecific [16]), but greater than those for aphids (0.24% intraspecific, 7.25% interspecific [12]). Among the 45 Japanese species treated by Kamitani [18] all species had distinct barcode sequences. By comparison, we found that 24% of species showed less than 1% minimum sequence divergence from their nearest-neighbour. For example, 11 closely related species in the leafhopper genus *Gyponana*, possessed pairwise interspecific distances ranging from 0 to 2.46% and a mean between-species divergence of only 1.38%. Despite their close sequence similarity, these species are distinguished morphologically by the form of the male genitalia, wing venation, and colour patterns, and exhibit biological differences [48]. These taxa likely represent instances of recent speciation through host specialization or geographic separation of populations with low vagility. The three species in the spittlebug genus *Philaenarcys* provide a similar example as they have different morphology, male genitalia and host plants [49], but there are only two sequence clusters, with specimens of *P. killa* in both. Various factors, including the retention of ancestral COI polymorphisms or mitochondrial introgression, may explain these situations, but in some cases they may also reflect the adoption of subtle inter-population differences as criteria for species delineation. Thus a reference library of DNA barcodes can motivate re-evaluation of the significance of morphological character differences among populations and species.

Pronotal shape has been an important character in defining some genera of Membracidae, such as *Telamona*. However, species of *Telamona* have barcodes similar to those of species of *Archasia*, *Carynota*, *Glossonotus* and *Heliria*. In fact, barcodes for specimens assigned to *Glossonotus acuminatus*, *Heliria cristata* and *Telamona concava* differ by less than 1%, a pattern which supports previous indications by morphological studies [50] that these genera need revision.

We detected a few examples of deep sequence divergence among specimens assigned to a single species (Table 3) suggesting the possibility of unrecognized cryptic species. For example, three specimens of *Carsonus furcatus*, all from Washington State, differ by up to 8.26%. Similarly, Mexican specimens currently assigned to *Cephisus variolosus* show divergences up to 7.5%. This species exhibits variation in shape and coloration, and Hamilton [51] has already suggested that it represents a complex of taxa. However, two specimens, collected together, of *Pagaronia minor*, a recently introduced Japanese species, diverged by about 4%. Sequences for these specimens are most similar to each other and distinct from those from any other species in the data set, so contamination is an unlikely explanation.

In general, broad geographic sampling results in an increase in observed intraspecific variation and a consequent decrease in minimum interspecific distance [52], [53]. The magnitude of this increase varies among taxa. All of the species treated in this barcode reference library are represented by few specimens and require further sampling from across their geographic ranges. This expanded data may result in an increase in the already rather high incidence of low interspecific divergenced in COI sequence among the Auchenorrhyncha. However, many species have quite restricted distributions, and additional sampling may reveal that for at least some of the species pairs with low divergence, intaspecific variation is limited and barcodes are truly diagnostic.

Further work is also necessary on many species groups to provide a more strongly validated taxonomic system to aid interpretation of COI sequence variation. Barcodes for type specimens would be especially valuable to correctly anchor the name associated with barcode clusters. Nevertheless, the utility of the method as a tool in the identification process and in species discovery was emphasized during the course of this study in that discrepancies in barcode sequence suggested errors in original species identification. On morphological re-examination the original determinations for 98 specimens were shown to be misidentifications, and in fact some of these specimens represent undescribed species. More than 3000 additional specimens from the CNC have now been sequenced and validation of these records and the identification of their source specimens are in progress.

### Data Availability Statement

Collection data, sequences, and trace files are available on BOLD (http://www.boldsystems) in public datasets DS-EMAUCH0 (primary dataset for Auchenorrhyncha, DOI: dx.doi.org/10.5883/DS-EMAUCH0) and DS-EMAUCH1 (short sequences, DOI: dx.doi.org/10.5883/DS-EMAUCH1).

## References

[1] Doering KC (1930) Synopsis of the family Cercopidae (Homoptera) in North America. J Kansas Entomol Soc 3: 53–108.

[2] Oman PW (1949) The Nearctic Leafhoppers (Homoptera: Cicadellidae): A Generic Classification and Check List. Mem Entomol Soc Washington 3: 1–253.

[3] Deitz LL, Wallace MS (2012) Richness of the Nearctic treehopper fauna (Hemiptera: Aetalionidae and Membracidae). Zootaxa 3423: 1–26.

[4] Sanborn AF, Heath MS (2012) The Cicadas (Hemiptera: Cicadoidea: Cicadidae) of North America North of Mexico. Thomas Say Publications in Entomology Monograph 32. 227 p.

[5] Nielson MW (1968) The leafhopper vectors of phytopathogenic viruses (Homoptera, Cicadellidae): taxonomy, biology, and virus transmission. USDA ARS Tech Bull 1382.

[6] Hamilton KGA (1985) Leafhoppers of ornamental and fruit trees in Canada/Cicadelles des arbres ornementaux et fruitiers du Canada. Agriculture Canada Publication 1779E/F. 71 p.

[7] Hamilton KGA (2005) Bugs reveal an extensive, long-lost northern tall-grass prairie. Bioscience 55: 49–59.

[8] Hamilton KGA, Whitcomb RF (2010) Chapter 8. Leafhoppers (Homoptera: Cicadellidae) a major family adapted to grassland habitats. *in:* Shorthouse JD, editor. Arthropods of Canadian Grasslands, 1: Ecology and Interactions in Grassland Habitats. Biological Survey of Canada Monograph Series No. 3. pp. 169–197.

[9] Nickel H, Hildebrandt J (2003) Auchenorrhyncha communities as indicators of disturbance in grasslands (Insecta, Hemiptera)—a case study from the Elbe flood plains (northern Germany). Agric Ecosyst Environ 98: 183–199 10.1016/S0167-8809(03)00080-X

[10] Hebert PDN, Cywinska A, Ball SL, deWaard JR (2003) Biological identifications through DNA barcodes. Proc R Soc Lond B Biol Sci 270: 313–322 10.1098/rspb.2002.2218 PMC1691236 12614582

[11] Floyd RM, Wilson JJ, Hebert PDN (2009) DNA barcodes and insect biodiversity. In: Foottit RG, Adler PH editors. Insect Biodiversity: Science and Society. Wiley-Blackwell, Oxford. pp 417–432.

[12] Foottit RG, Maw HEL, von Dohlen CD, Hebert PDN (2008) Species identification of aphids (Insecta: Hemiptera: Aphididae) through DNA barcodes. Mol Ecol Resour 8: 1189–1201 10.1111/j.1755-0998.2008.02297.x 21586006

[13] Lee W, Kim H, Lim J, Choi HR, Kim Y, et al (2010) Barcoding aphids (Hemiptera: Aphididae) of the Korean Peninsula: updating the global data set. Mol Ecol Resour 11: 32–37 10.1111/j.1755-0998.2010.02877.x 21429098

[14] Foottit RG, Maw HEL, Havill NP, Ahern RG, Montgomery ME (2009) DNA barcodes to explore diversity and life cycles in the Adelgidae (Insecta: Hemiptera: Aphidoidea). Mol Ecol Resour 9 Suppl 1: 188–195 10.1111/j.1755-0998.2009.02644.x 21564978

[15] Jung S, Duwal RK, Lee SH (2010) COI barcoding of true bugs (Insecta, Heteroptera). Mol Ecol Resour 11: 266–270 10.1111/j.1755-0998.2010.02945.x 21429132

[16] Park DS, Foottit RG, Maw HEL, Hebert PDN (2011) Barcoding bugs: DNA-based identification of the true bugs (Insecta: Hemiptera: Heteroptera). PLoS ONE 6 e18749 10.137/journal.pone.0018749 21526211 PMC3078146

[17] Park DS, Suh SJ, Hebert PDN, Oh HW, Hong KJ (2011) DNA barcodes for two scale insect families, mealybugs (Hemiptera: Pseudococcidae) and armored scales (Hemiptera: Diaspididae). Bull Entomol Res 101 (4) 429–34 10.1017/S0007485310000714 21272395

[18] Kamitani S (2011) DNA barcodes of Japanese leafhoppers. Esakia 50: 81–88.

[19] Cryan JR, Svenson GJ (2010) Family-level relationships of the spittlebugs and froghoppers (Hemiptera: Cicadomorpha: Cercopoidea). Syst Entomol 35: 393–415 10.1111/j.1365-3113.2009.00520.x

[20] Lin CP, Danforth BN, Wood TK (2004) Molecular phylogenetics and evolution of maternal care in membracine treehoppers. Syst Biol 53 (3) 400–421 10.1080/10635150490445869 15503670

[21] Ballman ES, Rugman-Jones PF, Stouthamer R, Hoddle MS (2011) Genetic structure of *Graphocephala atropunctata* (Hemiptera: Cicdellidae) populations across its natural range in California reveals isolation by distance. J Econ Entomol 104: 279–287 10.1603/EC10112 21404869

[22] Bennett GM, O'Grady PM (2012) Host–plants shape insect diversity: Phylogeny, origin, and species diversity of native Hawaiian leafhoppers (Cicadellidae: *Nesophrosyne*). Mol Phylogenet Evol 65: 705–717 10.1016/j.ympev.2012.07.024 22884527

[23] Hudson A, Richman DB, Escobar I, Creamer R (2010) Comparison of the feeding behavior and genetics of beet leafhopper, *Circulifer tenellus*, populations from California and New Mexico. Southwestern Entomol 35: 241–250 10.3958/059.035.0303

[24] Lee YJ, Hill KBR (2010) Systematic revision of the genus *Psithyristria* Stål (Hemiptera: Cicadidae) with seven new species and a molecular phylogeny of the genus and higher taxa. Syst Entomol 35: 277–305 10.1111/j.1365-3113.2009.00509.x

[25] Lin CP, Wood TK (2002) Molecular phylogeny of the North American *Enchenopa binotata* (Homoptera: Membracidae) species complex. Ann Entomol Soc Am 95 (2) 162–171.

[26] Lin CP, Cast MS, Wood TK, Chen MY (2007) Phylogenetics and phylogeography of the oak treehopper *Platycotis vittata* indicate three distinct North American lineages and a neotropical origin. Mol Phylogenet Evol 45: 750–756 10.1016/j.ympev.2007.06.005 17658274

[27] Marshall DC, Hill KBR, Cooley JR, Simon C (2011) Hybridization, mitochondrial DNA phylogeography, and prediction of the early stages of reproductive isolation: Lessons from New Zealand cicadas (Genus *Kikihia*). Syst Biol 60 (4) 482–502 10.1093/sysbio/syr017 21471306

[28] Marshall DC, Hill KBR, Marske KA, Chambers C, Buckley TR, et al (2012) Limited, episodic diversification and contrasting phylogeography in a New Zealand cicada radiation. BMC Evol Biol 12: 177 18 p. 22967046 10.1186/1471-2148-12-177 PMC3537654

[29] Maryańska-Nadachowska A, Drosopoulos S, Achowska DL, Kajtoch Ł, Kuznetsova VG (2010) Molecular phylogeny of the Mediterranean species of *Philaenus* (Hemiptera: Auchenorrhyncha: Aphrophoridae) using mitochondrial and nuclear DNA sequences. Syst Entomol 35: 318–328 10.1111/j.1365-3113.2009.00510.x

[30] Palomera V, Bertin S, Rodríguez A, Bosco D, Virla E, et al (2012) Is there any genetic variation among native Mexican and Argentinian populations of *Dalbulus maidis* (Hemiptera: Cicadellidae)? Fla Entomol 95: 150–155 10.1653/024.095.0123

[31] Shabani M, Bertheau C, Zeinalabedini M, Sarafrazi A, Mardi M, et al (2013) Population genetic structure and ecological niche modelling of the leafhopper *Hishimonus phycitis* . J Pest Sci 86: 173–183 DOI 10.1007s1034001204639/s10340-012-0463-9

[32] Smith PT (2005) Mitochondrial DNA variation among populations of the glassy-winged sharpshooter, *Homalodisca coagulata* . J Insect Sci 5 (41) 1–8. 17119623 10.1093/jis/5.1.41 PMC1615248

[33] de León JH, Fournier V, Hagler JR, Daane KM (2006) Development of molecular diagnostic markers for sharpshooters *Homalodisca coagulata* and *Homalodisca liturata* for use in predator gut content examinations. Entomol Exp Appl 119 (2) 109–119.

[34] Virant-Doberlet M, King RA, Polajnar J, Symondson WOC (2011) Molecular diagnostics reveal spiders that exploit prey vibrational signals used in sexual communication. Mol Ecol 20: 2204–2216 10.1111/j.1365-294X.2011.05038.x 21352388

[35] Le Roux JJ, Rubinoff D (2009) Molecular data reveals California as the potential source of an invasive leafhopper species, *Macrosteles* sp. nr. *severini*, transmitting the aster yellows phytoplasma in Hawaii. Ann Appl Biol 154: 429–439 10.1111/j.1744-7348.2008.00315.x

[36] Seabra SG, Pina-Martins F, Marabuto E, Yurtserver S, Halkka O, et al (2010) Molecular phylogeny and DNA barcoding in the meadow-spittlebug *Philaenus spumarius* (Hemiptera, Cercopidae) and its related species. Mol Phylogenet Evol 56: 462–467 10.1016/j.ympev.2010.03.023 20307671

[37] Metcalf ZP (1960–1962) General Catalogue of the Homoptera, fascicle 7, Cercopoidea [parts 1 to 4]. North Carolina State College.

[38] Hamilton KGA (1982) The Insects and Arachnids of Canada. Part 10. The Spittlebugs of Canada. Homoptera: Cercopidae. Agriculture Canada Publication 1740.

[39] Metcalf ZP (1946) General Catalogue of the Homoptera, fascicle IV, Fulgoroidea, 8. Dictyopharidae. Smith College, Birthampton, MA. 246 p.

[40] Metcalf ZP (1947) General Catalogue of the Homoptera, fascicle IV, Fulgoroidea, 9. Fulgoridae. Smith College, Birthampton, MA. 276 p.

[41] Ratnasingham S, Hebert PDN (2007) BOLD: The Barcode of Life Data System (www.barcodinglife.org). Mol Ecol Notes 7: 355–364 10.1111/j.1471-8286.2006.01678.x 18784790 PMC1890991

[42] Hajibabaei M, Singer GAC, Hebert PDN, Hickey DA (2007) DNA barcoding: how it complements taxonomy, molecular phylogenetics and population genetics. Trends Genet 23: 167172 10.1016/j.tig.2007.02.001 17316886

[43] Kimura M (1980) A simple method for estimating evolutionary rate of base substitutions through comparative studies of nucleotide sequences. J Mol Evol 16: 11–120. 7463489 10.1007/BF01731581

[44] Srivathsana A, Meier R (2011) On the inappropriate use of Kimura-2-parameter. Cladistics 28: 190–194 10.1111/j.1096-0031.2011.00370.x 34861755

[45] Collins RA, Boydkin LM, Cruickshank RH, Armstrong KF (2012) Barcoding's next top model: an evaluation of nucleotide substitution models for specimen identification. Methods Ecol Evol 3: 457–465 10.1111/j.2041-210X.2011.00176.x

[46] Tamura K, Peterson D, Peterson N, Stecher G, Nei M, et al (2011) MEGA5: Molecular Evolutionary Genetics Analysis using Maximum Likelihood, Evolutionary Distance, and Maximum Parsimony Methods. Mol Biol Evol 28 (10) 2731–2739 10.1093/molbev/msr121 21546353 PMC3203626

[47] Consortium for the Barcode of Life (2005) Data Standards for BARCODE Records in INSDC (BRIs). Available: http://barcoding.si.edu/PDF/DWG_data_standards-Final.pdf. Accessed 2013 Nov 8.

[48] Hamilton KGA (1982) Review of the Nearctic species of the nominate subgenus of *Gyponana* Ball (Rhynchota: Homoptera: Cicadellidae). J Kan Entomol Soc 55: 547–562.

[49] Hamilton KGA (2011) What we have learned from shutterbugs. American Entomol 57: 102–109.

[50] Wallace MS (2011) Morphology-based phylogenetic analysis of the treehopper tribe Smiliini (Hemiptera: Membracidae: Smiliinae), with reinstatement of the tribe Telamonini. Zootaxa 3047: 1–42.

[51] Hamilton KGA (2012) Revision of Neotropical aphrophorine spittlebugs, part 1: Pteylini (Hemiptera, Cercopoidea). Zootaxa 3497: 41–59. 10.11646/zootaxa.3710.3.1 26106685

[52] Bergsten J, Bilton DT, Fujisawa T, Miranda E, Monaghan M, et al (2012) The Effect of Geographical Scale of Sampling on DNA Barcodingl Syst Biol. 61: 851–869 10.1093/sysbio/sys037 PMC3417044 22398121

[53] Zahiri R, Lafontaine JD, Schmidt BC, deWaard JF, Zakharov V, et al (2014) A Transcontinental Challenge - A Test of DNA Barcode Performance for 1541 Species of Canadian Noctuoidea (Lepidoptera). PLoS ONE in press. 10.1371/journal.pone.0092797 PMC3965468 24667847

[54] Hebert PDN, Penton EH, Burns JM, Janzen DH, Hallwachs W (2004) Ten species in one: DNA barcoding reveals cryptic species in the neotropical skipper butterfly *Astraptes fulgerator* . Proc Natl Acad Sci USA 101: 14812–14817 10.1073/pnas.0406166101 15465915 PMC522015

[55] Folmer O, Black M, Hoeh W, Lutz R, Vrijenhoek R (1994) DNA primers for amplification of mitochondrial cytochrome c oxidase subunit I from diverse metazoan invertebrates. J Exp Mar Bio Ecol 3 (5) 294–299. 7881515

[56] Park DS, Suh SJ, Oh HW, Hebert PDN (2010) Recovery of the mitochondrial COI barcode region in diverse Hexapoda through tRNA-based primers. BMC Genomics 11: 243 12 p. 20615258 10.1186/1471-2164-11-423 PMC2996951

